# Metaplastic carcinoma of the breast with transformation from adenosquamous carcinoma to osteosarcomatoid and spindle cell morphology

**DOI:** 10.3892/ol.2013.1464

**Published:** 2013-07-15

**Authors:** SUEBWONG CHUTHAPISITH, MALEE WARNNISSORN, NATTAWUT AMORNPINYOKIAT, KANAPON PRADNIWAT, TAMNIT ANGSUSINHA

**Affiliations:** 1 Department of Surgery, Faculty of Medicine Siriraj Hospital, Mahidol University, Bangkok 10700, Thailand; 2 Department of Pathology, Faculty of Medicine Siriraj Hospital, Mahidol University, Bangkok 10700, Thailand; 3 Thanyarak Breast Imaging Center, Faculty of Medicine Siriraj Hospital, Mahidol University, Bangkok 10700, Thailand

**Keywords:** adenosquamous, breast, metaplastic carcinoma, osteosarcoma, spindle cell

## Abstract

Metaplastic carcinoma of the breast refers to a heterogenous group of mammary carcinomas that contain a mixture of various cell types, including squamous cells, spindle cells and/or a mesenchymal component, such as bone or cartilage. To the best of our knowledge, the clinical course of a tumour that has undergone a transformation from one type of metaplastic carcinoma to another subtype has not previously been reported. The present study reports the five-year clinical and pathological course of a metaplastic breast carcinoma in a 55-year-old female, who was diagnosed with a sclerosing fibroadenomatous nodule with osseous metaplasia and focal atypia. A recurrent tumour was documented four years later, showing a predominant component of osteosarcoma with adenosquamous carcinoma. Upon pathological review of the initial mass, the diagnosis was changed to low-grade adenosquamous carcinoma. The patient was treated with breast conserving therapy. However, one year later, a recurrent metaplastic carcinoma with spindle cell morphology was documented and surgically removed by mastectomy. Subsequently, pulmonary invasion of the chest wall occurred and the patient eventually succumbed due to the invasive nature of the disease.

## Introduction

Breast cancer is the most common cancer that affects females worldwide, with >1.3 million cases newly diagnosed each year. Amongst all breast cancers, 85% are invasive ductal carcinomas. The metaplastic subtype is unusual. According to the published study, based on the United States National Cancer Database, of the 365,484 breast cancers that were diagnosed between 2001 and 2003, 892 were metaplastic carcinomas (1). Thus, the incidence was 0.24%. Metaplastic carcinoma is pathologically characterized by a mixture of epithelial and mesenchymal neoplasms. Osseous and cartilaginous changes are frequently observed in the mesenchymal change (2). Osseous metaplasia of metaplastic breast cancer is relatively rare, accounting for <0.5% of all breast cancers (2–4). The treatment of metaplastic carcinoma of the breast is inconclusive due to the rarity of this subtype. The prognosis of the disease is worse than that of invasive ductal carcinoma (5–7). The present study reported the clinical and pathological course of a case of metaplastic breast cancer with transformation from a low-grade adenosquamous carcinoma to an osteosarcomatoid component with spindle cell morphology. Written informed consent was obtained from the patient.

## Case report

**Figure 1:**
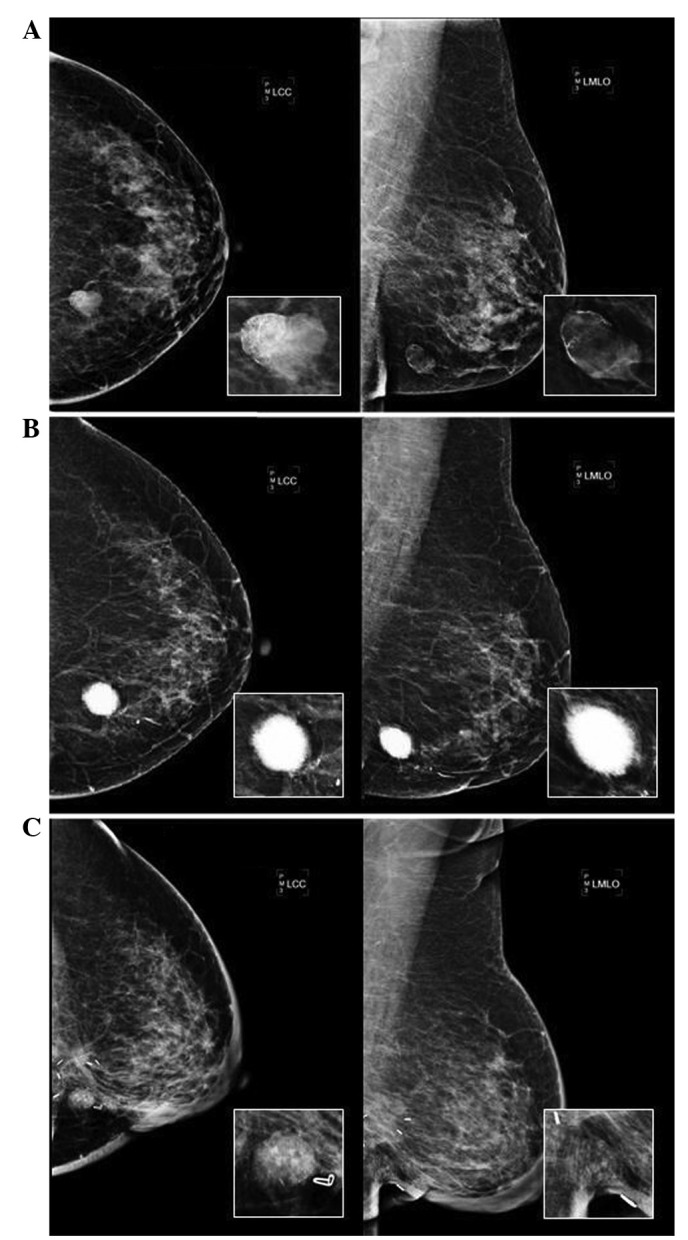
(A) Mammography performed in 2005. CC and MLO views show an oval, well-circumscribed mass, isodensity with a rim and calcification. (B) Mammography performed in 2009. CC and MLO views show a large calcified mass at the left inferior inner quadrant. The magnified pictures reveal the sunburst appearance, which resembled osteosarcoma calcification. (C) Mammography performed in 2010. CC and MLO views show a well-defined 1.5-cm mass with multiple coarse calcifications underneath the surgical scar. CC, cranio-caudal; MLO, medio-lateral oblique.

In 2005, a 55-year-old female presented with a lump in the inferior inner quadrant of the left breast that had been present for 3 years. Mammography revealed a 2-cm partially rim-calcified mass with a well-defined border. Additional ultrasonography showed a well-defined, hypoechoic calcified mass (Fig. 1A). The mass was excised for pathological examination and showed a sclerosing fibroadenomatous nodule with focal atypia and osseous metaplasia. Following the excision, clinical examination and imaging identified no further significant abnormalities.

**Figure 2:**
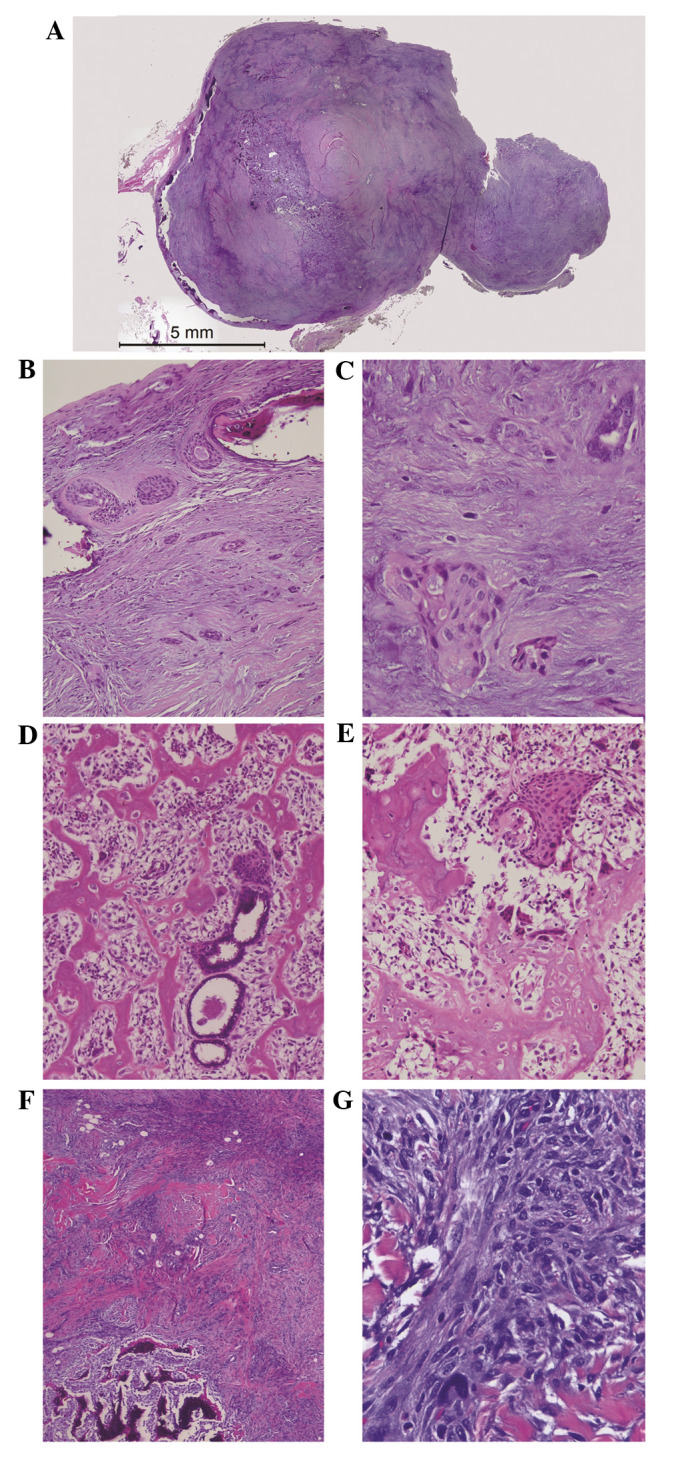
(A–C) First mass removed in 2005. (A) Low-grade adenosquamous carcinoma with focal osseous metaplasia, initially diagnosed as nonmalignant fibroadenomatous nodule (H&E). (B) Presence of banal-appearing epithelial component in fibromyxoid stroma (H&E; magnification, ×200). (C) Low-grade angulated tubules and irregular squamous nests (H&E; magnification, ×400). (D and E) Recurrent tumor in 2009. (D) Prominent osteosarcomatous component with few entrapped glands (H&E; magnification, ×200). (E) Entrapped squamous epithelium (H&E; magnification, ×200). (F and G) Recurrent tumor in 2010. (F) Focal osseous component (H&E; magnification, ×40). (G) Predominant spindle cell morphology without any epithelial cells documented (H&E; magnification, ×400). H&E, hematoxylin and eosin.

In 2009, a lump was observed on the left breast of the patient underneath the previous surgical site. Mammography revealed a 2.8-cm sunburst calcified mass, mimicking osteosarcoma, at the inferior inner area of the left breast, corresponding to the palpable area (Fig. 1B). The mass was surgically removed and appeared circumscribed but focally infiltrative microscopically. Histologically, the tumour appeared to contain an extensive osteosarcomatous part with minor epithelial components (<5%). The latter component consisted of scattered small glands, elongated tubules and cell nests showing low grade atypia, rare mitosis and occasional squamous differentiation. They were randomly distributed in the osteosarcomatous part (Fig. 2D and E) and in the collagenous stroma adjacent to the mass. Immunohistochemical staining revealed that the luminal cells were stained more intensely by CK5/6 and CK7, but that the remaining areas were stained weakly or displayed no staining. Certain single layer cords and tubules also displayed staining. The tumour showed inconsistent staining with p63 and calponin from absent, attenuated or complete circumferential staining. These findings supported the diagnosis of metaplastic carcinoma showing low-grade adenosquamous carcinoma and with osteosarcomatous components. Review of the initial mass excised in 2005 disclosed the deceptive low-grade adenosquamous carcinoma (Fig. 2A–C), in part resembling the epithelial component in the recurrent mass. The tumour was negative for the oestrogen receptor (ER), progesterone receptor (PR) and HER-2 (score, 1+). Due to the focal invasion at the surgical margin, a removal of the additional margin and a sentinel lymph node biopsy were performed. Pathologically, the cavity and the sentinel lymph nodes contained no tumour. Therefore, according to the TNM classification, the tumour was T2N0M0, stage IIa. Following the surgery, adjuvant doxorubicin and cyclophosphamide was administered for four courses with 50-Gy whole breast irradiation.

In 2010, a 1.5-cm mass size was detected underneath the surgical scar. The mammogram and ultrasound showed a poorly-defined border mass (Fig. 1C). A recurrence of metaplastic cancer at the previous tumour bed was confirmed following the biopsy (Fig. 2F and G). The patient underwent a modified radical mastectomy. The pathological assessment reported metaplastic carcinoma with spindle cell morphology in two foci (1.2 and 0.8 cm, respectively), adjacent to the previous surgical site (Fig. 2C). All surgical margins were free with no axillary lymph node involvement. Further immunohistochemical staining showed no staining for ER and HER-2, but weak staining (10–25%) for PR. The patient was administered oral tamoxifen at 20 mg per day.

At four months after the mastectomy, recurrent nodules at the surgical scar were observed, as well as multiple pulmonary nodules that were compatible with pulmonary metastasis. A histological analysis revealed a diagnosis of recurrent spindle cell metaplastic carcinoma (Fig. 2D). The patient was prescribed with carboplatin and paclitaxel for two cycles. However, the chemotherapy was not well-tolerated and was ceased. The patient was lost to follow-up and eventually succumbed due to the invasive nature of the disease.

## Discussion

Metaplastic carcinoma was first described in 1973 and was denoted as a mammary carcinoma with mixed epithelial and sarcomatoid components (8). The classification of metaplastic carcinoma is mainly based on histological findings of purely epithelial (squamous, adenosquamous and spindle cell carcinomas) and mixed epithelial and mesenchymal (carcinoma with chondroid/osseous metaplasia and carcinosarcoma) components (9).

Metaplastic carcinoma usually involves females aged >50 years, with a rapidly growing palpable mass and bloody nipple discharge (7,8,10). The patient from the present study was 55 years old, which is consistent with this previously established age range. Diagnosing metaplastic carcinoma using breast imaging is challenging, since only the benign features of the disease are observed. The typical malignant characteristics of breast cancer that are visualized using mammography, including pleomorphic and linear microcalcification, are not frequently documented in metaplastic carcinoma. Instead, a round or oval mass with a circumscribed margin are described (11). Furthermore, ultrasonographic findings are not useful, since an oval, round or lobular solid hypoechoic mass with circumscribed or indistinct margins are observed (10,11). As a result, the lesion may be easily misinterpreted and that may delay the diagnosis (11). In the present study, a notable point in the mammography that was performed in 2009 was the sunburst calcification, which resembled osteosarcoma and was observed in the cranio-caudal (CC) and medio-lateral oblique (MLO) views (Fig. 1B).

Upon microscopic examination, a diagnosis becomes apparent, with the exception of certain instances. Metaplastic carcinoma, including low-grade adenosquamous carcinoma, may deceptively appear indistinguishable from a benign proliferative breast lesion. Certain metaplastic carcinomas may exhibit minimal epithelial components compared with the mesenchymal component. Others may exhibit a virtually complete replacement of the adenocarcinoma with the metaplastic element (5,12). Immunohistochemical staining is useful in highlighting the epithelial component and determining the invasion and evaluation of the mesenchymal component.

The pathological findings in the present study raised certain notable concerns. The first tumour in 2005 (Fig. 2A) was diagnosed as a sclerosing fibroadenomatous nodule with osseous metaplasia and focal atypical epithelium, which was benign. However, upon review of the histology, the diagnosis was revised to a low-grade adenosquamous carcinoma with osseous metaplasia. The deceptive features that favoured the benign appearance were low-grade nuclei and sparsely scattered small tubules. Certain neoplastic ducts were difficult to distinguish from the entrapped normal ducts at the periphery. The fibrocollagenous stroma that contained scattered ducts relatively resembled a sclerosing lesion. The features that favoured the malignant nature included the disarrayed distribution of angulated tubules and certain single cells with no discernible basement membrane, compared with the prominent basement membrane and lobulocentric pattern of the sclerosing adenosis or the zonal pattern of the radial scar. Immunohistochemical staining was not discriminatory due to an inconsistent pattern of staining, as described previously (13). Negative staining for ER contradicted the presence of a benign lesion and low-grade carcinoma, but indicated the presence of a metaplastic low-grade adenosquamous carcinoma (9).

In the present study, within the series of microscopic assessments of the first tumour in 2005 until the fourth tumour in 2010, a transformation was observed from a low-grade adenosquamous carcinoma to a carcinosarcoma showing a predominant osteosarcomatous component and minor adenosquamous carcinoma in the second tumour, and then finally a metaplastic carcinoma with spindle cell morphology (Fig. 2A–D). To the best of our knowledge, this is the first study to describe the transformation of one type of metaplastic carcinoma to another.

Osseous metaplasia is an unusual condition that is reported in non-neoplastic, benign and malignant conditions of the breast. Osseous metaplasia of benign breast tumours has been reported in fibroadenoma, mammary pleomorphic adenoma, benign mesenchymoma, phyllodes tumours and primary localized amyloid tumours (14,15). Osseous metaplasia of malignant neoplasms may occur from epithelial or mesenchymal tissue. The majority of neoplasias are derived from mesenchymal tissue, including fibrosarcoma, malignant mesenchymoma, osteoid sarcoma, osteogenic sarcoma, osteochondrosarcoma and fibromatosis. In non-neoplastic tissues, osseous metaplasia is associated with chronic mastitis, a possible ossified hematoma, pseudohypoparathyroidism and myositis ossification. Three possible hypotheses have been described with regard to metaplasia of stromal origin: i) Ossification associated with calcific debris; ii) metaplasia without antecedent stromal changes; and iii) metaplasia in areas of tumour mucin secretion (16).

The majority of studies describe the presence of a large tumour during the diagnosis of metaplastic carcinomas, possibly due to a more rapid growth (1,5,17). Patients with metaplastic carcinoma of the breast are usually associated with poorly-differentiated or undifferentiated tumours (7,12,17,18), suggesting a reason for the aggressive behaviour of the tumour, and a large and rapidly-growing mass at presentation (1). Thus, a tumour of >5 cm in size is considered to predict a poor outcome (11).

In the present study, the disease became recurrent following a negative sentinel lymph node biopsy and modified radical mastectomy. However, the tumour did not exhibit axillary metastasis. Axillary metastasis has previously been observed to be less frequent in metaplastic carcinoma of the breast compared with ductal carcinoma (7). The incidence of axillary lymph node metastasis at diagnosis has been shown to vary between 11.8 and 53.0% (1,7,11,12,17,18). However, metaplastic carcinomas have demonstrated a high potential for distant metastases, particularly in the lung, even if the lymph nodes are negative (6). Clinical evidence of pulmonary metastasis was noted in the present case.

Metaplastic breast carcinomas are usually high-grade and negative for hormonal receptors (82–100%) (1,5,6,12,17–19). The absence of the predominant glandular epithelial component may also explain the rarity of ER/PR expression. Nuclear staining may present in the area of ductal differentiation, but not in areas of spindle cell or squamous cell carcinoma (17). Metaplastic breast carcinoma appears to have little HER-2/neu overexpression, with a positive rate of 7–14%, unlike other high-grade breast carcinomas, which have a HER-2 positivity rate of 25–30% (5,12,17,18), suggesting that metaplastic carcinoma may have an alternative biology to high-grade invasive ductal carcinoma (18). ER, PR and HER-2 negativity is regarded as ‘triple negative’ breast cancer, which tends to have a poor prognosis. However, in a small study on the prognosis of metaplastic carcinoma, patients with non-triple negative metaplastic carcinoma had poorer prognoses than those with triple-negative breast cancer (20).

Patients with metaplastic breast carcinoma may be treated with breast conserving surgery. However, this is less frequent than for patients with ductal carcinoma (1,5,7,19), due to a large tumour size at presentation (1). Apart from surgery, adjuvant post-operative treatment for metaplastic carcinoma is similar to the treatment for invasive ductal carcinoma, but it lacks sufficient data in terms of effectiveness. In a study of a small number of patients with metaplastic carcinoma, seven of nine patients who received adjuvant chemotherapy (adriamycin/cyclophosphamide, cyclophosphamide/methotrexate/5-fluorouracil and cyclophosphamide/adriamycin/5-fluorouracil regimens) developed recurrent disease (6), which is consistent with the patient of the present case study. Thus, the standard regimen for ductal carcinoma may not be effective for metaplastic breast carcinoma. Patients with metaplastic carcinoma who receive adjuvant chemotherapy have demonstrated a better survival rate than those who do not (5). However, due to the small number of cases that occur, it is difficult to perform further cohort randomized controlled trials.

Compared with ductal carcinoma, metaplastic breast carcinoma tends to have a worse outcome, with a disease-free survival rate of 40–62% and an overall survival rate of 40–73% (6,7). However, when the nodal status and tumour size were adjusted in a small series from the Memorial Sloan-Kettering cancer center, more favorable disease-free and overall survival rates were observed (19).

In conclusion, metaplastic carcinoma with transformation from one subtype to another is a rare condition. Confirming a diagnosis is difficult and requires various histological studies. The behavior of the tumour is likely to be more aggressive than that of a ductal carcinoma. The tumour commonly presents as a large mass with less nodal involvement, a higher tumour grade and a lack of hormonal and HER-2 receptors. The principles of treatment for metaplastic carcinoma are the same as those for invasive ductal carcinoma, but recurrence is more likely, as shown in the present case study.
